# Facile Green Synthesis of Iron Oxide Nanoparticles and Their Impact on Cytotoxicity, Antioxidative Properties and Bactericidal Activity

**DOI:** 10.61186/ibj.4061

**Published:** 2024-01-15

**Authors:** Sejal S. Shah, Bhavika P. Turakhia, Nihar Purohit, Khushal M. Kapadiya, Chita R. Sahoo, Akhilesh Prajapati

**Affiliations:** 1 Department of Bioinformatics, Faculty of Technology, Marwadi University, Rajkot, Gujarat, India;; 2 Department of Life Sciences, Biotechnology Division, School of Science, GSFC University, Vadodara-391750, Gujarat, India;; 3 School of Science, Department of Chemistry, RK University, Rajkot, Gujarat, India;; 4 School of Chemistry, Cardiff University, Cardiff Wales, CF 10 3AT, United Kingdom;; 5 Central Research Laboratory, Institute of Medical Sciences & SUM Hospital, Siksha O Anusandhan Deemed to be University, Bhubaneswar 751003, Odisha, India

**Keywords:** Antioxidants, Bactericidale activity, Green Synthesis, MDA-MB-231cells, Molecular docking

## Abstract

**Background:**

Bioreductive processes are quite potent, effective and affordable for the synthesis of green NPs, as compared to the physical and chemical methods. The present study aimed to evaluate the bactericidal, antioxidative and anticancer activity of FeONPs derived from the turmeric rhizome (*Curcuma amada*) using ferric chloride as a precursor.

**Methods:**

With focusing on the manufacture of FeONPs via green approach, we characterized the NPs using FTIR, FT-Vis, DLS, and UV-Vis spectroscopy. The produced particles were tested for antibacterial, antioxidant, and anticancer properties. The synthesized NPs were also examined using the MDA-MB-231 human epithelial breast cancer cell line and NCI-60 cancer cell lines.

**Results:**

The antioxidant activity of TR-FeONPs was concentration-dependent. The scavenging activity of TR-FeONPs was 76.09% at a concentration of 140 µg/ml. Using different concentrations of TR-FeONPs in the MTT assay against the MDA-MB-231 cell line indicated a reduction of less than 50% in cell viability at 125 µg/ml. Moreover, TR-FeONPs exhibited an effective bactericidal property. The gTR-FeONPs synthesized bioreductively were found to be effective in renal cancer, UO-31 cell line, with GI_50_ value of 66.64%.

**Conclusion:**

Our study showcases a sustainable method based on green chemistry principles to produce FeONPs utilizing turmeric rhizome. We anticipate that the FeONPs produced through this biosynthesis process could serve as a promising drug delivery system in cancer treatment and as an effective antimicrobial agent against various diseases.

## INTRODUCTION

Bioreductive synthesis of NPs mediated by the plant is a potent method, playing a vital role in the field of modern medicine^[^^1^^]^. In comparison to the production of bulk particles, NPs unveil novel and enhanced properties based on their characteristics such as size, distribution, morphology, and phase^[^^2^^]^. There are various techniques for the synthesis of metal NPs; however, eco-friendly synthesizing NPs without using hazardous chemicals is of great importance^[^^3^^]^. Biotic methods, e.g. the use of microorganism, enzymes, and plant extracts, are considered an environment-friendly alternative for the synthesis of NPs^[^^4^^,^^5^^]^. Due to the large surface area to volume ratio, NPs have vast application in the area of waste water treatment^[^^2^^]^, pharmaceutics, textile coating technology, pharmacogenomics^[^^3^^-^^5^^]^, cancer treatment^[^^6^^]^, and drug delivery^[^^7^^]^.

Turmeric possesses antioxidant, anticancer, anti-inflammatory, and bactericidal properties due to the presence of various phytochemicals such as curcumin, dimethoxy curcumin, and bisdemethoxycurcumin, as well as sugars, resins, proteins, and essential oils such as turmerone, atlantone, and zingberone, which altogether are called curcuminoid. Curcumin is the most potent antimicrobial and antioxidant agent found in tumeric^[^^8^^,^^9^^]^. Curcumin is soluble in ethanol (10 mg/ml), DMSO (25 mg/ml), acetic acid, NaOH (0.1 M), Na_2_CO_3_ (10 mM), acetone, water (<0.1 mg/ml), and chloroform but insoluble in cold water, cellulose, and gum^[^^10^^]^. NPs of expensive metals, such as gold, silver, and platinum, have also been shown to have excellent antimicrobial, antioxidant, and anticancer activities^[^^11^^-^^13^^]^.

The present study was conducted with focusing on the synthesis of FeONPs, as it is eco-friendly, inexpensive, and non-toxic and also a favorable alternative to expensive metals such as gold and silver. Herein, we synthesized FeONPs via a green chemistry approach, followed by the characterization of FeONPs using UV-Vis spectroscopy, XRD, FTIR, and DLS. Furthermore, the synthesized particles were evaluated for bactericidal, antioxidant, and anticancer activities. As this research was a pilot study, the synthesized NPs were investigated on a specific cell line, MDA-MB-231, an epithelial human breast cancer cell line, using MTT assay. Further, FeONPs were analyzed against NCI-60 cancer cell lines. The bioactive compounds of *C. amada *were subjected to molecular docking to assess the antimicrobial activities. The current investigation highlights the potentiality of phytochemicals present in turmeric to reduce and stabilize Fe^+3^ ions for the synthesis of FeONPs.

## MATERIALS AND METHODS

**Preparation of plant extract**

Turmeric rhizome (*Curcuma amada*) also known as turmeric or amba haldar belongs to the Zingiberaceae family. The plant was collected from the herbal garden of the School of Science, RK University (Gujarat, India) in November 2018 and identified by a botanist from the above-mentioned University. The plant sample was deposited at the herbarium of the university with reference no. RKU/SOS/HB/001/2019. The rhizome was dried, and 6 g of its powder was added to 100 ml of methanol in a conical flask. The solution was stirred for an hour using a magnetic stirrer (Remi Electrotechnics Limited, India) at 60 °C. The extract was then filtered and stored at 4 °C before use^[^^14^^]^.

**Preparation of FeONPs**

FeCl_3 _98% was purchased from Molychem (India) and used without further purification. FeCl_3_ solution (0.01 M) was added to the turmeric extract in a 2:3 volume ratio. The solution was stirred in a magnetic stirrer at 70 °C for 5 h. After the color change was observed, the precipitate was resuspended in distilled water and centrifuged immediately. The suspension was dehydrated in hot air oven at 50 °C to attain NPs in powdered form^[^^15^^,^^16^^]^.

**Characterization of turmeric NPs**

To confirm the formation of NPs, different characterization methodologies such as UV-Vis spectroscopy, SEM, DLS, and XRD, were used to determine their size, shape, morphology and structure. The bioreduction of Fe^+3^ was monitored by a UV-Vis spectrophotometer (Labtronics–Lt 2900, India)^[^^16^^]^. FTIR of FeONPs was recorded over a range of 650–4,000 cm^-1^ with a resolution of 4 cm^-1^ on FTIR spectrometer (Agilent Cary 630, California, USA). The crystallographic structure, chemical composition, and physical properties of materials and thin films were characterized by XRD (PANalytical X’Pert Pro X-ray diffractometer at the Department of Physics, Saurashtra University, Rajkot). DLS was utilized to measure particle size distribution^[^^17^^,^^18^^]^.

**Bactericidal studies**

Antimicrobial activity of FeONPs was determined using agar well diffusion method^[^^18^^]^. Gram-positive (*B. subtilis *MCC2244 and *S. aureus *MCC2043) and Gram-negative (*E. coli *MCC2246) bacteria were activated in a nutrient broth overnight. The efficacy of synthesized FeONPs (10 mg/ml) was evaluated against penicillin (10 mg/ml), distilled water, turmeric rhizome extract, and FeCl_3_ (0.01 M). All the agar plates were incubated at 37 °C for 24 hours, and the zone of inhibition was observed^[^^18^^]^. 

**Antioxidant studies**

Evaluation of free radical scavenging activity was determined by DPPH assay. DPPH is a crystalline purple color substance that accepts hydrogen ions from an antioxidant agent, which in turn shows the color change from purple to yellow. FeONPs (20-100 µg/ml concentrations) were added to 5 ml of 0.1 mM of ethanolic DPPH solution. The mixture was stored in the dark for 30 min, and the absorbance was measured at 517 nm. The DPPH solution without the addition of NPs was regarded as a control. The antioxidant activity of synthesized FeONPs was compared with the plant extract and ascorbic acid as the standards^[^^19^^] ^and calculated by using the following formula:

$\mathrm{Scavenging activity}(\%)=\frac{\mathrm{Absorbance oƒ control}–\mathrm{Absorbance oƒ sample}}{\mathrm{Absorbance oƒ controle}}\times 100$

**In vitro** **cytotoxicity assay against NCI-60 cell line**

**Fig. 1:**
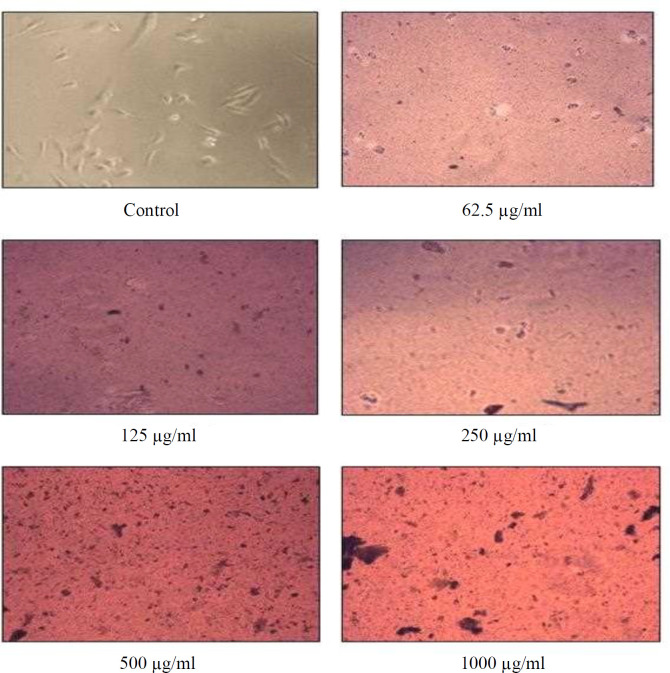
Morphological changes in MDA-MB-231 cells following treatment with different concentrations of TR-FeONPs

Cytotoxic activity of the synthesized FeONPs was performed at National cancer Institute/National Institutes of Health in Bethesda, Maryland, USA.The outline of the study can be observed at https://dtp.cancer.gov. FeONPs were tested in MDA-MB-231 cells, and then 100 µl of media were seeded at the optimal density of ~10,000 cells per well in 96-well plates, which were kept in a 5% CO_2_ incubator at 37 °C overnight. After reaching 40-50% confluence, the cells were treated in triplicate with different concentrations of the synthesized FeONPs (Fig. 1). Following 48 hours, MTT solution (5 mg/ml) was added to the cell culture medium. After incubating t*he 96-well plate in a 5% *CO_2 _*incubator at 37 °C for 3 h, we observed formazan crystals under a microscope. The media was handled with utmost care to prevent any harm to the developing formazan crystals. Subsequently, the crystals were dissolved in DMSO with a volume of 100-200 µl per well. The 96-will plate was then placed on a shaker in a dark environment for a period of 10 minutes, and the absorbance at 570 nm was examined*^[^^20^^-^^22^^]^*.*

**Molecular docking studies**

The bioactive compounds of *C. amada*, as shown in Table 1, were retrieved from online PubChem Database (https://pubchem.ncbi.nlm.nih.gov/). Each structure of biological active compounds of *C. amada* was neatly drawn by Chem Draw Ultra12.0 using stereo optimization. Subsequently, crystal structure of bacterial protein, namely DNA gyrase of *S. aureus *(PDBID: 3G7B), Glyoxal reductase of *B. subtilis *(PDBID: 3D3F), and DNAgyrase of *E. coli* (PDBID: 1 KZN), were retrieved from protein databank (https://www. rcsb.org/)^[^^23^^]^. Molecular docking studies were carried out by AutoDock tool, and visualization of protein-ligand interactions was performed by Biovia Discovery Studio 2017.

**Table 1:** Molecular docking score of bioactive compounds from *C. amada*

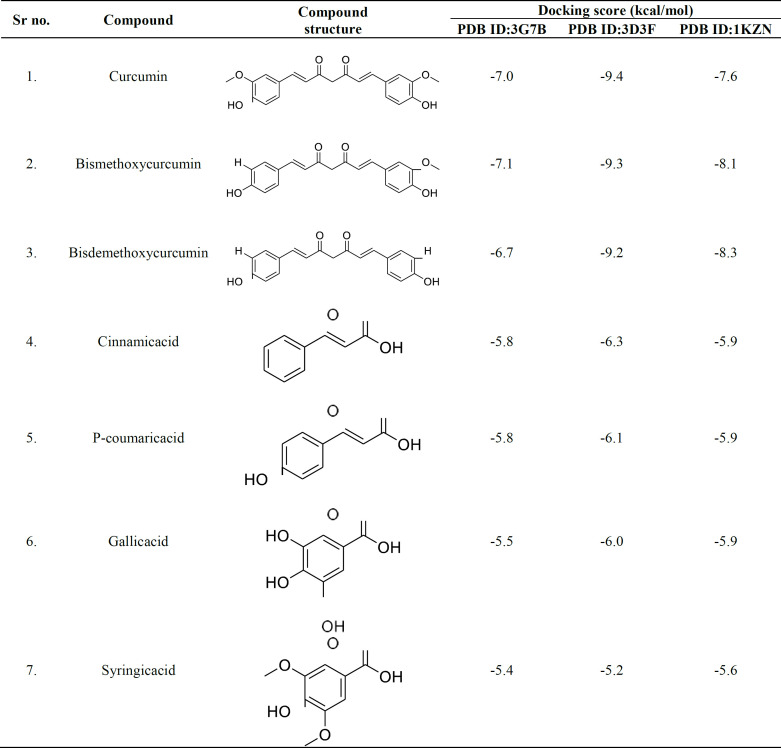

**Statistical analysis**

SPSS 15.0 was employed for statistical analysis of all the data. One way analysis of variance (ANOVA) was used to statistically analyze the inhibition zone of various bacteria. *P* value less than 0.05 was considered as statistically significant.

## RESULTS

**Primary indication of formation of FeONPs**

**Fig. 2:**
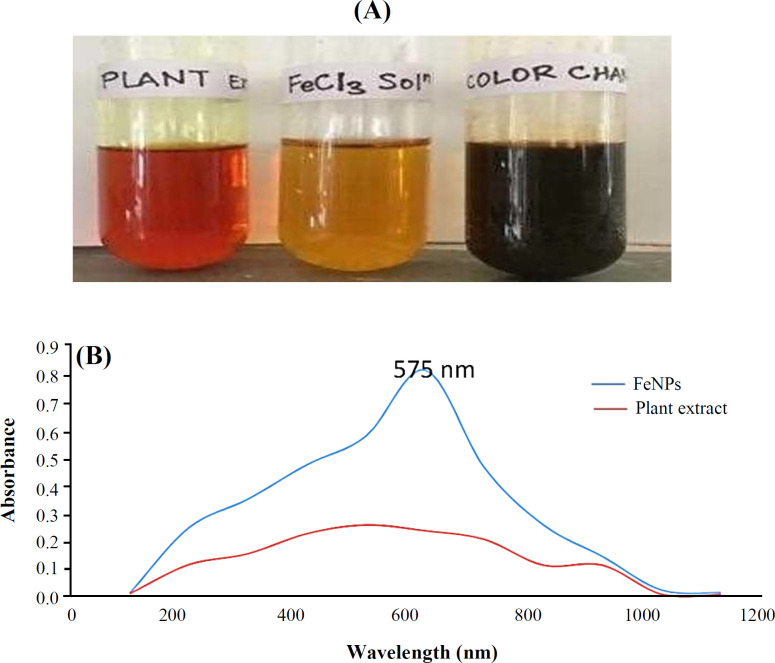
(A) Visual inspection of color change for the formation of FeONPs; (B) UV-Vis spectroscopy of FeONPs and plant extract

The color change is shown in Figure 2A, in which the solution of plant extract was observed as dark orange, and when FeCl_3 _was added to the solution, it turned to dark brown due to reduction of Fe^3+ ^to Fe^0^, confirming the results of previous studies^[^^23^^-^^25^^]^.

**Characterization of synthesized FeONPs**

**Fig. 3:**
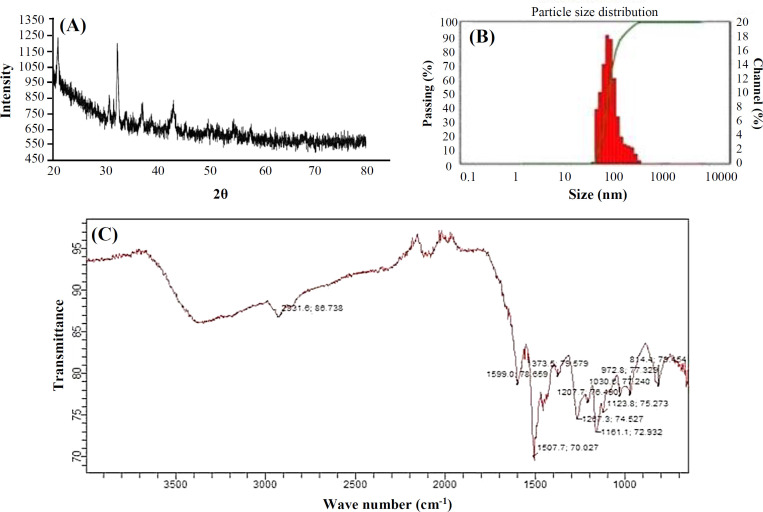
(A) XRD analysis and (B) particles size distribution, and (C) FTIR analysis of TR-FeONPs

The UV-Vis spectrum of aqueous turmeric extract and the synthesized NPs is illustrated in Figure 2B. The aqueous turmeric extract peak was observed to be between 200 and 300 nm, whereas the synthesized NPs speak was found to be between 400 and 500 nm. The XRD spectrum of the synthesized FeONPs is depicted in Figure 3A. The sharp peak at 32.6° and 36.7° indicated the presence of iron oxide, whereas the peak at 44.7º was attributed to orthorhombic structure of Fe. Characterization of the functional groups in turmeric rhizome and the synthesized FeONPs was conducted through FTIR analysis. This analysis helps in elucidating the surface functional groups involved in interactions with metals. The shift in bond stretching from 3512.37 to 3431 cm^-1^ (-OH bond stretching), 2945.30 to 2931 cm^-1^ (-CH_3_ stretching), 1280.75 to 1267.3 cm^-1^ (amide bond stretching), from 1627.92 to 1599 cm^-1 ^(-COO bond stretching), and from 1025 to 1030 cm^-1 ^(CN bond stretching) was observed. However, comparing turmeric rhizome extract with TR-FeONPs indicated the formation of TR-FeONPs^[^^26^^]^. The mean size of particles in the solution was measured using DLS (Fig. 3B).

**Bactericidal activity of TR-FeONPs**

**Fig. 4:**
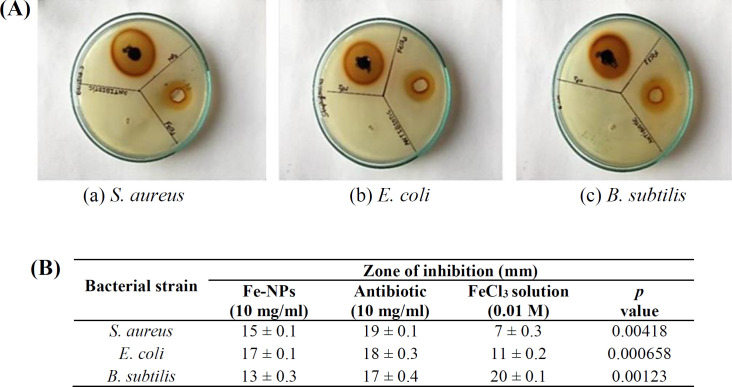
(A) Antimicrobial activity of synthesized TR-FeONPs; (B) statistical analysis

**Fig. 5:**
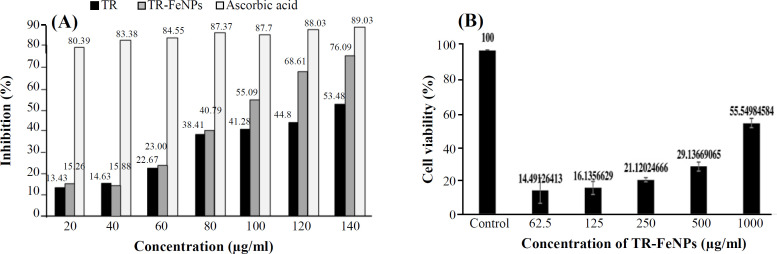
(A) Antioxidant activity of TR-FeONPs; (B) MTT cell viability assay against MDA-MB-231 cell line

The bactericidal activity of the synthesized TR-FeONPs was tested against Gram-positive (*B. subtilis *MCC2244 and *S. aureus *MCC2043) and Gram-negative (*E. coli *MCC2246) bacteria, and penicillin was used as the standard. The inhibition zone of FeONPs was compared with the positive control, plant extract, and 0.01 M of FeCl_3_, as illustrated in Figure 4. The turmeric rhizome extract with a concentration of 10 mg/ml demonstrated a zone of inhibition due to the presence of curcuminoids and various other phytochemicals. The synthesized TR-FeONPs (10 mg/ml) showed a higher zone of inhibition than the turmeric rhizome extract (10 mg/ml), as depicted in Figure 4B. The size of the NP and high surface area to volume ratio might be the reason for the higher bactericidal activity of TR-FeONPs than TR extract. The TR-FeO NPs exhibited potential promising ability to scavenge free radicals (Fig. 5A). As the concentration of NPs increased from 20 µg/ml to 140 µg/ml, the scavenging activity also enhanced from 15.26% to 76.09%.

**Cytotoxicity assay**

**Fig. 6:**
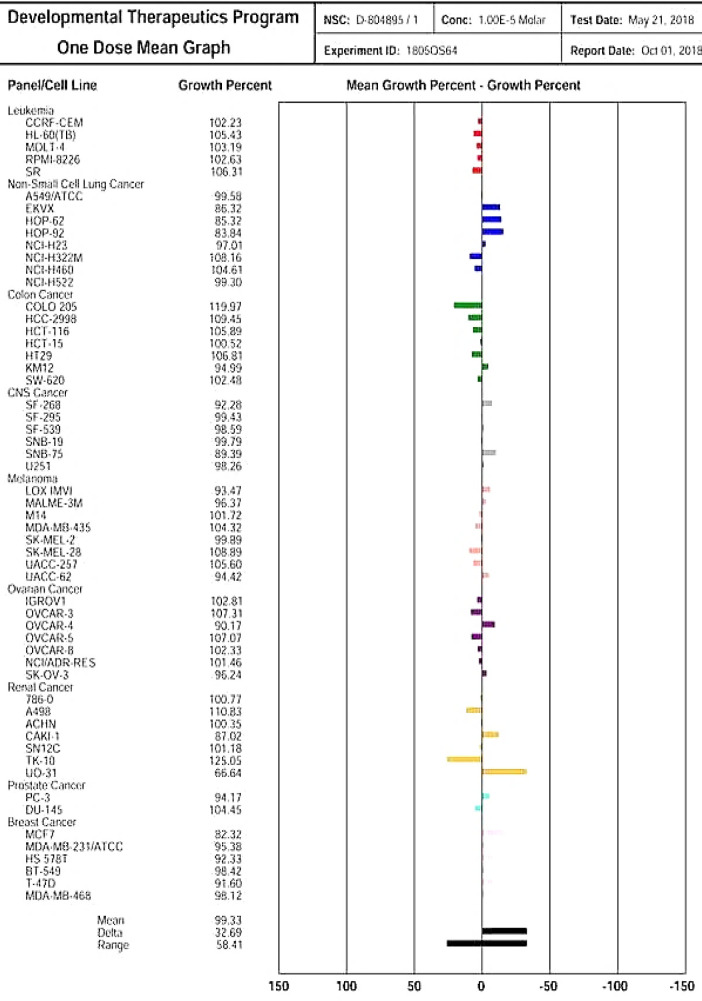
Anti-cancer dose-response graph of iron oxide NPs against NCI-60 cell line

The MTT assay revealed cytotoxicity for TR-FeONPs against the MDA-MB-231 cell line. A considerable decrease in the viability of MDA-MB-231 cell line was observed after treatment with the synthesized TR-FeONPs. With increasing the concentration of the NPs, viability decreased. Complete mortality was observed, as depicted in Figure 5B. Iron oxides are potential candidates in cancer therapy because of their super paramagnetic behavior and surface modification characteristics^[^^27^^,^^28^^]^. Biosynthesized TR-FeNP was evaluated against NCI-60 cancer cell lines in nine distinct cancer panels, comprising various cell lines, followed by single dose response study (Fig. 6)^[^^27^^,^^28^^]^. The findings undeniably indicated that renal cancer exhibited the highest GI50 value among all other cancer types. In renal cancer, the UO-31 cell line showed remarkable anticancer activity with a growth rate of 66.64%. Among all the selected cells, the non-small cell lung cancer demonstrated moderate activity with GI_50_ values of 80-100%. Hence, our study suggests that the derived FeONOs show a favorable outcome toward renal cancer cells.

**In silico** **study**

**Fig. 7:**
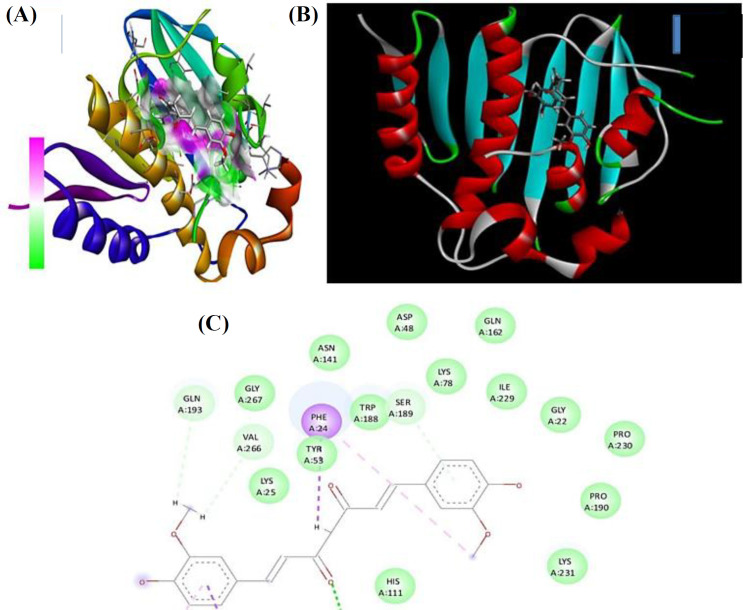
Crystal structure of bacterial protein. (A) DNA gyrase of *S. aureus *(PDBID: 3G7B); (B) glyoxal reductase of *B. subtilis *(PDBID:3D3F); (C) DNA gyrase of* E. coli *(PDBID:1KZN). In the binding process between the lead compound of curcumin and glyoxal reductase of *B. subtilis* H-bond donors and acceptors play a crucial role. The ligand, with its three-dimensional geometric structure, occupies the binding site of the protein. The binding interactions of amino acid residues occur in a two-dimensional fashion

In silico investigation was carried out to identify the potential inhibitory action of bioactive compounds of*C. amada against *bacterial proteins. Curcumin and bismethoxy curcumin exhibited the lowest binding energy, -7.0, -9.4, and -7.6 kCal/mol against 3G7B, 3D3F, and 1KZN, respectively. In general, glyoxal reductase of *B. subtili*s and curcumin from turmeric is a potent inhibitor, which interacts with amino acid residues Trp122, Pro268, Tyr53, and Phe24. DNA gyrase is an essential enzyme in bacteria that plays a crucial role in DNA replication and maintenance. DNA gyrase involves in various aspects of DNA metabolism, including replication, transcription, and maintaining the structural integrity of the bacterial genome. Its essential role in these processes makes it a target for antibiotics and a key player against bacterial cell viability. Molecular docking study is depicted in Figure 7 and Table 1.

## DISCUSSION

The field of cancer nano-therapeutics is advancing rapidly and is being utilized to address various disadvantages of traditional drug delivery systems. These drawbacks include non-specific distribution and targeting, limited water solubility, low oral bioavailability, and low therapeutic indices. To enhance the distribution of cancer drugs, NPs have been engineered with optimal size and surface properties to prolong their presence in the bloodstream.

The eco-friendly and effective method of plant-mediated green synthesis of NPs has found significant application in the medical field. Current investigation involves cost effective, ecofriendly green synthesis of FeONPs from turmeric rhizome. Confirmation of the synthesized FeONPs involves different characterization methods. In UV-Vis spectroscopy, the absorption peak was observed in the formation of FeONPs at the wavelength of 400-600 nm. The sharp peak at 420 nm was due to the surface plasmon resonance of FeONPs. The UV-Vis spectrum of the synthesized FeONPs was similar to that reported by Sankar et al.^[^^29^^]^. The diffraction peak of XRD at 2θ values of 32.6°, 36.7°, and 44.7° corresponded to the crystallane (110), (111), and (311) of FeONPs. The same as our previous reports, resultant NPs were α-Fe2O3 with orthorohombic structure^[^^30^^,^^25^^]^. The size distribution of FeONPs synthesized from turmeric was in the range of 30-170 nm with an average size of 60 nm.

In our findings, TR-FeONPs showed higher bactericidal activity than the plant extract. The smaller size of NPs may be the key characteristic of TR-FeONPs. As these NPs can easily penetrate into the cell wall of bacteria, they show efficient bactericidal activity. The current study revealed a higher zone of inhibition (17 mm ± 0.1) for the *E. coli *MCC2246 strain than *B. subtilis *MCC2244 and *S. aureus *MCC2043. The zone of inhibition outcomes for *S. aureus* MCC2043, *B. subtilis* MCC2244, and *E. coli* MCC2246 revealed *p* values of 0.00418, 0.00123, and 0.000658, correspondingly. Given that the *p* values are less than 0.05, the null hypothesis is accepted, suggesting a significant variance in the zone of inhibition.

The variation in the composition of the cell wall between Gram-positive and Gram-negative bacteria could potentially have a significant impact on the zone of inhibition^[^^18^^,^^31^^-^^33^^]^. The present study identified that antioxidant activity of TR-FeONPs is concentration-dependent. At a concentration of 140 µg/ml, TR-FeONPs exhibited a scavenging activity of 76.09%, comparable to ascorbic acid, a standard antioxidant. This outcome suggests the potential use of the synthesized TR-FeONPs as an alternative antioxidant for diseases linked to reactive oxygen species.

Various concentrations of TR-FeONPs were tested against the MDA-MB-231 cell line using MTT assay. The concentration of 125 µg/ml resulted in a decreased cell viability by less than 50%. The cytotoxicity mechanism of TR-FeONPs may involve triggering apoptosis in tumor cells by enhancing the production of reactive oxygen species. Furthermore, TR-FeONPs were assessed on the NCI-60 cell line, with renal cancer showing the highest GI50 value compared to other cancer cell lines/panels. According to the computational analysis, it is evident that the glyoxal reductase of *B. subtilis*, when combined with curcumin, exhibits promising inhibitory properties. This inhibition is attributed to the interaction between curcumin and specific amino acid residues, including Trp122, Pro268, Tyr53, and Phe24. 

## CONCLUSION

This study presents a bioreductive, eco-friendly, simple, and cost-effective method for synthesizing FeONPs using turmeric rhizome plant extract. The synthesized TR-FeONPs exhibit promising bactericidal activity against human pathogens and also possess nano-antioxidative properties. These features hold potential in the field of medicine for treating diseases related to the scavenging of free radicals. The study recognizes the constraints of in vivo experiments in assessing the accuracy and cytotoxic effects of TR-FeONPs on tumor and normal cells. By utilizing the fabrication process to develop smart nanoparticles, their interaction capacity with cells can be improved, leading to increased cytotoxicity and reduced side effects. Molecular docking analysis of the phytocompounds present in *C. amada*, including curcumin and bismethoxycurcumin, revealed their potent phytochemicals with antimicrobial activities, indicating their potential usefulness in future pharmacological applications.

## DECLARATIONS

### Acknowledgments

Authors has not used any AI technology in generation of current research work.

### Ethical approval

Not applicable.

### Consent to participate

Not applicable.

### Consent for publication

All authors reviewed the results and approved the final version of the manuscript.

### Authors’ contributions

SSS: conceptualization, methodology, software; BPT: data curation, writing-original draft preparation; NP: visualization and investigation; CRS: computational analysis and molecular docking; KMK: software and validation; AP: manuscript writing- reviewing and editing and data discussion.

### Data availability

All relevant data can be found within the manuscript. 

### Competing interests

The authors declare that they have no competing interests. 

### Funding

This research received no specific grant from any funding agency in the public, commercial, or not-for-profit sectors.

### Supplementary information

The online version does not contain supplementary material. 
